# Alteration of Gut Microbiota Composition and Diversity in Acute and/or Chronic Graft-versus-Host Disease Following Hematopoietic Stem Cell Transplantation: A Prospective Cohort Study

**DOI:** 10.3390/ijms25115789

**Published:** 2024-05-26

**Authors:** Eleni Gavriilaki, Maria Christoforidi, Konstantinos Ouranos, Fani Minti, Despina Mallouri, Christos Varelas, Andriana Lazaridou, Eirini Baldoumi, Alkistis Panteliadou, Zoi Bousiou, Ioannis Batsis, Ioanna Sakellari, Georgia Gioula

**Affiliations:** 1 2nd Propedeutic Department of Internal Medicine, Aristotle University of Thessaloniki, 54643 Thessaloniki, Greece; 2 Microbiology Department, Aristotle University of Thessaloniki, 54124 Thessaloniki, Greece; mhrist@auth.gr (M.C.); fani.minti@yahoo.com (F.M.); ggioula@auth.gr (G.G.); 3 Department of Medicine, Houston Methodist Research Institute, Houston, TX 77030, USA; kouranos@houstonmethodist.org; 4 Hematology Department—BMT Unit, G Papanikolaou Hospital, 57010 Thessaloniki, Greece; dmallouri@gmail.com (D.M.); varelaschris@gmail.com (C.V.); andrianalazaridou@gmail.com (A.L.); eir_balntou@yahoo.gr (E.B.); kirapanteliadou@gmail.com (A.P.); boussiou_z@hotmail.com (Z.B.); iobats@yahoo.gr (I.B.); ioannamarilena@gmail.com (I.S.)

**Keywords:** gut microbiota, graft-versus-host disease, stem cell transplantation, microbial diversity, microbial abundance

## Abstract

Changes in gut microbiome composition have been implicated in the pathogenesis of graft-versus-host disease (GvHD) after allogeneic hematopoietic stem cell transplantation (allo-HSCT). Our objective was to explore the microbial abundance in patients with GvHD after allo-HSCT. We conducted a single-center, prospective study in patients who underwent allo-HSCT and developed grade II or higher acute GvHD and/or moderate or severe chronic GvHD, to explore the microbial abundance of taxa at the phylum, family, genus, and species level, and we utilized alpha and beta diversity indices to further describe our findings. We collected fecal specimens at −2 to +2 (T1), +11 to +17 (T2), +25 to +30 (T3), +90 (T4), and +180 (T5) days to assess changes in gut microbiota, with day 0 being the day of allo-HSCT. We included 20 allo-HSCT recipients in the study. Compared with timepoint T1, at timepoint T4 we found a significant decrease in the abundance of *Proteobacteria* phylum (14.22% at T1 vs. 4.07% at T4, *p* = 0.01) and *Enterobacteriaceae* family (13.3% at T1 vs. <0.05% at T4, *p* < 0.05), as well as a significant increase in *Enterococcus* species (0.1% at T1 vs. 12.8% at T4, *p* < 0.05) in patients who developed acute GvHD. Regarding patients who developed chronic GvHD after allo-HSCT, there was a significant reduction in the abundance of *Eurobactereaceae* family (1.32% at T1 vs. 0.53% at T4, *p* < 0.05) and *Roseruria* genus (3.97% at T1 vs. 0.09% at T4, *p* < 0.05) at T4 compared with T1. Alpha and beta diversity analyses did not reveal a difference in the abundance of bacteria at the genus level in GvHD patients at T4 compared with T1. Our study reinforces results from previous studies regarding changes in gut microbiota in patients with acute GvHD and provides new data regarding the gut microbiome changes in chronic GvHD. Future studies will need to incorporate clinical parameters in their analyses to establish their association with specific changes in gut microbiota in patients with GvHD after allo-HSCT.

## 1. Introduction

Allogeneic hematopoietic stem cell transplantation (allo-HSCT) is a curative treatment modality for hematologic malignant and non-malignant disorders [1]. Still, allo-HSCT is accompanied by significant morbidity and mortality, primarily due to complications, including graft-versus-host disease (GvHD) [2] and infections [3]. In GvHD, grafted immunocompetent T cells proliferate in the immunocompromised host and attack recipient cells, leading to severe organ dysfunction [4].

Recent insights into the development of GvHD after allo-HSCT have emphasized the role of microbiome in pathogenesis and treatment-related outcomes [5]. Following a conditioning regimen, damage to the mucosal epithelial surfaces causes gut bacterial translocation and altered microbial homeostasis [6]. Moreover, gut decontamination through prophylactic antibiotic administration can profoundly affect the composition of aerobic and anaerobic intestinal flora which have been shown to be involved in the pathogenesis of GvHD [7,8]. Both the type of antibiotic administered, either broad- or narrow-spectrum [9], and the timing of administration, that is, before, during, or after allo-HSCT [10], have been associated with microbiota diversity loss and variable long-term clinical outcomes. Studies harnessing high-throughput sequencing methods to characterize gut microbial structure and diversity before and after allo-HSCT have established the impact of dysbiosis on GvHD development and mortality [11,12,13]. 

Although most studies contemplate the association between microbiota composition and acute GvHD (aGvHD) after allo-HSCT, the exact perturbations in gut microbial structure and diversity are not yet completely elucidated. Moreover, the exact gut microbiome composition in patients with chronic GvHD (cGvHD) has not been analyzed. In an effort to characterize the changes occurring in microbiota composition of patients with aGvHD and cGvHD, we conducted a prospective study in patients undergoing allo-HSCT. 

## 2. Results

### 2.1. Patient Characteristics

During the study period, 44 patients underwent allo-HSCT. Two patients declined study participation and 22 patients did not have enough samples collected to run the analysis of fecal specimens. As such, 20 patients were included in the study. In Table 1, we present baseline characteristics of patients included in the analysis. Briefly, we included 8 patients with aGvHD (median age 50 years) and 12 patients with cGvHD (median age 52.6 years). The most common underlying hematologic disease in patients with aGvHD was acute myeloid leukemia (*n* = 3) and acute lymphoblastic leukemia (*n* = 3), and in patients with cGvHD, the most common diagnosis was acute myeloid leukemia (*n* = 5). Patients with aGvHD presented with grade II (*n* = 3) and grade III (*n* = 5) disease, while cGVHD was moderate in the majority of patients (*n* = 8) and severe in four patients. The mean hematopoietic cell transplantation–comorbidity index (HCT-CI) was 2.3 and 2.6, and the mean EBMT score was 3.5 and 3.6 in patients with aGvHD and cGvHD, respectively. 

Acute GvHD involved the gastrointestinal tract in four out of eight (50%) patients with aGvHD, whereas skin involvement was present in two out of eight (25%) patients with aGvHD. Acute GvHD did not resolve but was controlled under treatment. Moderate or severe cGvHD involved the skin, lungs, and liver in the majority of patients, specifically 10 out of 12 (83.3%) patients with cGvHD. Chronic GvHD remained stable after treatment in the majority of patients with cGvHD, specifically 7 out of 12 (58.3%), while downgrading to moderate occurred in the remaining patients with cGvHD, specifically 5 out of 12 (41.7%).

### 2.2. Abundance Analysis Results

We explored the data at the phylum, family, genus, and species levels, by applying the abundance analysis. For each level, we reported all the taxa detected per timepoint and then we compared their abundance to detect statistically significant differences. A summary of the sequencing and microbiome profiling is provided in Supplementary Table S1.

Among patients that developed aGvHD, abundance analysis at the phylum level revealed a statistically significant reduction in the abundance of the *Proteobacteria* phylum, from 14.22% at timepoint 1 to 4.07% at timepoint 4 (*p* = 0.01), while changes in the total abundance of the other phyla were not statistically significant. At the family level, *Enterobacteriaceae* family abundance was reduced from 13.3% at timepoint 1 to <0.05% at timepoint 4 (*p* < 0.05). Regarding the genus level, an abundance of *Enterococcus* species was increased from 0.1% at timepoint 1 to 12.8% at timepoint 4 (*p* < 0.05). Finally, at the species level, no statistically significant differences were observed in the abundance of the studied species. We present the most abundant taxa per level in timepoint 1 and 4 for aGvHD, in Table 2 and Supplementary Figures S1–S4.

Regarding patients that developed cGvHD, no significant differences in the abundance at the phylum and species levels were found between timepoint 1 and timepoint 4. At the family level, there was a significant reduction in the abundance of *Eurobactereaceae* family, from 1.32% at timepoint 1 to 0.53% at timepoint 4 (*p* < 0.05). At the genus level, the abundance of *Roseruria* genus was significantly reduced, from 3.97% at timepoint 1 to 0.09% at timepoint 4 (*p* < 0.05). We present the most abundant taxa per level in timepoint 1 and 4 for cGvHD, in Table 3 and Supplementary Figures S5–S8.

### 2.3. Alpha Diversity Analysis Results

**Figure 1:**
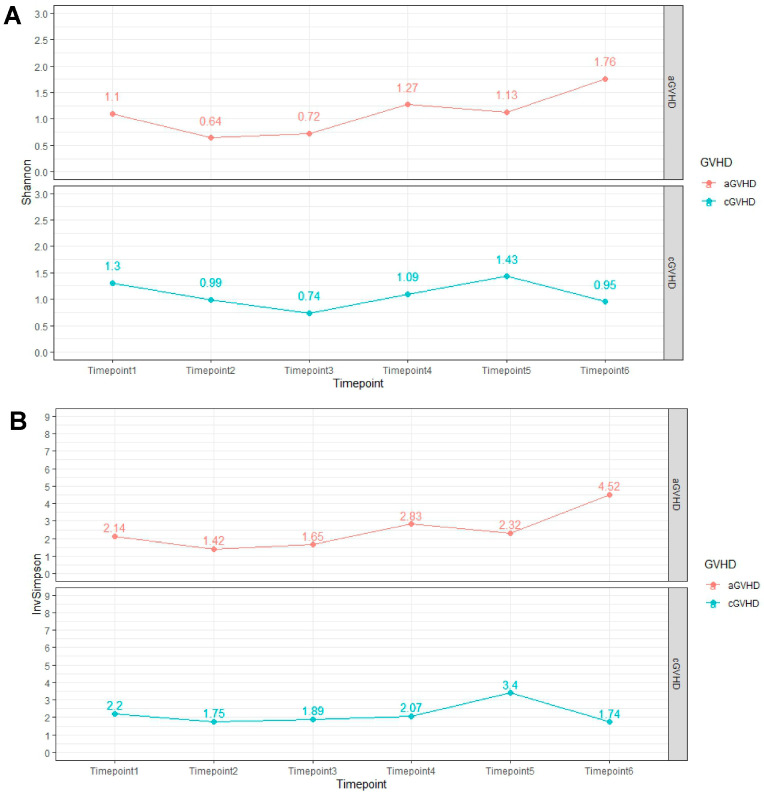
(**A**). Alpha diversity analysis using the Shannon index at the genus level across the pre-defined study timepoints in patients with aGVHD and cGVHD. Abbreviations list: aGVHD: acute graft-versus-host disease; cGVHD: chronic graft-versus-host disease; (**B**). Alpha diversity analysis using the inverse Simpson index at the genus level across the pre-defined study timepoints in patients with aGVHD and cGVHD. Timepoints T1, T2, T3, T4, and T5 correspond to −2 to +2, +11 to +17, +25 to +30, +90, and +180 days following stem cell transplantation. Median values are presented in the figures. Abbreviations list: aGVHD: acute graft-versus-host disease; cGVHD: chronic graft-versus-host disease.

No significant associations were observed in alpha diversity analysis, including the Shannon index (*p* = 0.45) and inverse Simpson index (*p* = 0.2), at the genus level from timepoint 1 to timepoint 4 in patients with aGvHD (Figure 1A, Supplementary Table S2). 

Likewise, alpha diversity analysis did not reveal a statistically significant difference for both the Shannon index (*p* = 0.64) and inverse Simpson index (*p* = 0.86) regarding abundance at the genus level from timepoint 1 to timepoint 4 in patients with cGvHD (Figure 1B, Supplementary Table S2).

### 2.4. LEfSe Analysis Results

**Figure 2:**
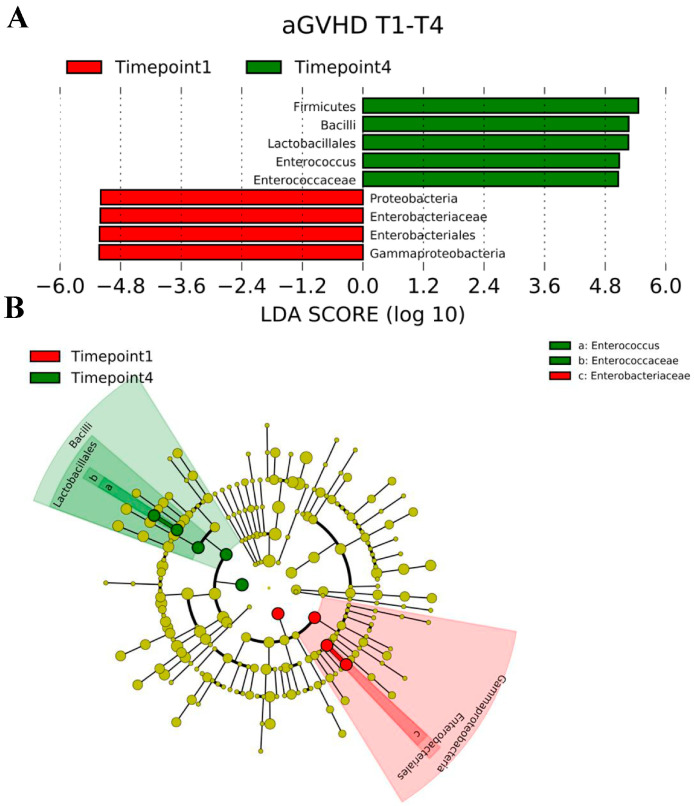
(**A**). LDA coupled with effect size measurements identifies *Firmicutes*, *Bacilli*, *Lactobacillales*, *Enterococcus*, and *Enterococcaceae* as significantly enriched taxa at timepoint 4, while the abundance of *Proteobacteria*, *Enterobacteriaceae*, *Enterobacteriales*, and *Gammaproteobacteria* is significantly reduced at timepoint 1 in patients with aGVHD. (**B**). Cladogram generated by LEfSe indicating differences in the abundance of taxa between timepoint 1 and timepoint 4 in patients with aGVHD. Each successive circle represents a phylogenetic level (phylum, family, genus, species). Regions in green indicate taxa enriched during timepoint 4, while regions in red indicate a reduction in the abundance of taxa during timepoint 1. *Bacilli*, *Lactobacillales*, *Enterococcus*, and *Enterococcaceae*, were significantly enriched at timepoint 4, while the abundance of *Gammaproteobacteria*, *Enterobacteriales*, and *Enterobacteriaceae* was significantly reduced at timepoint 1 Abbreviations list: aGVHD: acute graft-versus-host disease; LEfSe: Linear discriminant analysis Effect Size.

We performed LEfSe analysis to generate a histogram of the LDA score and a cladogram to identify the specific microbiome involved in GvHD. LDA distribution diagram analysis (LDA score > 2) showed an increase in the abundance of *Firmicutes*, *Bacilli*, *Lactobacillales*, *Enterococcus*, and *Enterococcaceae* at timepoint 4 (*p* < 0.05), while the abundance of *Proteobacteria*, *Enterobacteriaceae*, *Enterobacteriales*, and *Gammaproteobacteria* was significantly reduced at timepoint 1 (*p* < 0.05) (Figure 2A). From the cladogram, we showed that, in patients with aGvHD, *Bacilli*, *Lactobacillales*, *Enterococcus*, and *Enterococcaceae*, were significantly enriched at timepoint 4 (*p* < 0.05), while the abundance of *Gammaproteobacteria*, *Enterobacteriales*, and *Enterobacteriaceae* was significantly reduced at timepoint 1 (*p* < 0.05) (Figure 2B). 

**Figure 3:**
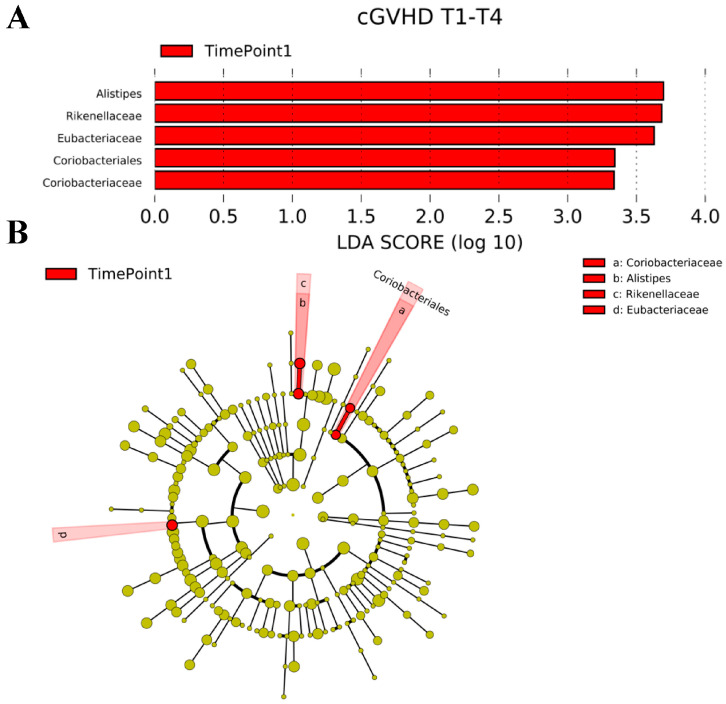
(**A**). LDA coupled with effect size measurements indicates that the abundance of *Alistipes*, *Rikinellaceae*, *Eubacteriaceae*, *Coiobacteriales* and *Coriobacteriaceae* at timepoint 1 is significantly reduced in patients with cGVHD. (**B**). Cladogram generated by LEfSe indicating differences in the abundance of taxa at timepoint 1 in patients with cGVHD. Each successive circle represents a phylogenetic level (phylum, family, genus, species). Regions in red indicate reduction in the abundance of taxa at timepoint 1. The abundance of *Coriobacteriaceae*, *Alistipes*, *Rikenellaceae*, and *Eubacteriaceae* was significantly reduced at timepoint 1. Abbreviations list: cGVHD: chronic graft-versus-host disease; LEfSe: Linear discriminant analysis Effect Size.

Regarding patients with cGvHD, LDA distribution diagram analysis (LDA score > 2) showed a significant reduction in the abundance of Alistipes, Rikinellaceae, *Eubacteriaceae*, *Coiobacteriales* and *Coriobacteriaceae* at timepoint 1 (*p* < 0.05) (Figure 3A). Likewise, the cladogram showed that the abundance of Coriobacteriaceae, Alistipes, Rikenellaceae, and *Eubacteriaceae* was significantly reduced at timepoint 1 (*p* < 0.05), with no significant differences observed at the phylum, family, genus, or species level at timepoint 4 (Figure 3B).

## 3. Discussion

In this study, we aimed to characterize the changes in the microbiota composition occurring in patients who develop GvHD after allo-HSCT. From our results, we found a significant decrease in the abundance level of the Proteobacteria phylum and Enterobacteriaceae family, as well as a significant increase in the Enterococcus species in patients who developed aGvHD after allo-HSCT compared to the microbiota composition examined during the time of allo-HSCT. Regarding patients that developed cGvHD after allo-HSCT, there was a significant reduction in the abundance of Eurobactereaceae family and Roseruria genus compared to the microbiota composition examined during the time of allo-HSCT. Alpha and beta diversity analyses did not reveal a difference in the abundance of bacteria at the phylum, family, genus, or species level in patients who developed GvHD after allo-HSCT compared to the microbiota composition examined during the time of allo-HSCT.

Although high-dose glucocorticoids are typically harnessed as first-line treatment for GvHD, the high failure rate, approaching 60% in patients with grade IV disease [14], necessitates the development of new targeted therapies [15]. The treatment armamentarium of GvHD has expanded over the past few years, with clinical trials currently underway to assess the effectiveness and safety of new regimens [16,17]. Fecal microbiota transplantation has also been studied for the treatment of GvHD, given the pivotal role of the gut microbiome in the pathogenesis of the disease [18]. Further insights into the association of the gut microbial community with GvHD may provide us with novel clues regarding disease severity and prognosis. 

In our study, the Enterococcus species showed a significant increase in abundance after allo-HSCT in patients who developed aGvHD. The expansion of this species has also been validated in previous studies assessing microbial perturbations in patients after allo-HSCT [19,20,21]. In murine GvHD models, enterococcal domination increased the number of donor T-cells and led to the recruitment of CD4+ T-cells and T helper 17 (Th17) cells in the lamina propria, all of which have been implicated in the pathogenesis of GvHD [22,23]. Regarding clinical implications, abundance of Enterococcus has been associated with severe GvHD, worse survival after allo-HSCT [24], and increased risk of enterococcal bacteremia [25]. The observed increase in the abundance of Enterococcus may be attributed to the translocation of the bacterium because of the disruption of the mucosal epithelial barrier, which is caused by structural alterations of the gut mucosa and depletion of commensal gut microbes in the setting of chemoradiotherapy and prophylactic antibiotic administration in allo-HSCT [5,9]. The choice of antibiotic therapy also seems to influence the abundance of Enterococcus, since a study conducted by Weber et al. in patients undergoing allo-HSCT revealed that the use of rifaximin correlated with lower enterococcal positivity compared to patients receiving ciprofloxacin/metronidazole [26]. We did not correlate our results with the specific type of antibiotic administered. In the future, prospective studies on the role of Enterococcus on GvHD severity and outcomes after allo-HSCT should incorporate the choice of antibiotic therapy in their analyses to delineate the impact of specific antibiotic regimens in the abundance of enterococci. 

Next, our study revealed a decrease in the abundance level of the Proteobacteria phylum and Enterobacteriaceae family. Contrary to our findings, studies published in the literature have suggested that Proteobacteria and Enterobacteriaceae bloom in patients with aGvHD after allo-HSCT [27,28,29]. Bacteria belonging to the Enterobacteriaceae family promote Th17-mediated inflammatory responses that have been implicated in the pathogenesis of aGvHD [9,30]. Even more, Han et al. [29] reported that Enterobacteriaceae negatively correlate with the regulatory T cell (Treg)/Th17 cell ratio, suggesting that the balance between Tregs, which attenuate inflammatory responses [31], and Th17 cells, which promote inflammation, is modified by intestinal microbiota, thus influencing the development of aGvHD. 

In our analysis, diversity indices did not reveal any differences in the abundance of bacteria at the genus levels 90 days after allo-HSCT compared to the time during allo-HSCT. The lack of significant findings may be attributed to the fact that we included a small number of patients in our analysis. Nevertheless, loss of gut microbial diversity has been described in the literature in patients undergoing allo-HSCT, with lower diversity levels correlating with increased overall mortality. Specifically, Taur et al. collected fecal specimens from 80 patients undergoing allo-HSCT and by performing metagenomic analyses, they revealed that low diversity groups had worse 3-year overall survival compared to patients in the high and intermediate diversity groups [10]. Similar findings were reported by Peled et al., who studied gut microbial disruptions in patients undergoing allo-HSCT at four different institutions and showed that loss of diversity and domination by single taxa was associated with increased mortality compared to patients with higher diversity [13]. Loss of commensal gut microbes after allo-HSCT has been attributed to the conditioning regimen and type of antibiotic prophylaxis received, leading to intestinal epithelial cell disruption, domination by pathologic bacteria, immune cell infiltration and establishment of the infectious milieu observed in GvHD [32,33]. As a result, microbial diversity can be used as a prognostic marker to identify patients undergoing allo-HSCT who are at high risk of developing GvHD. 

We also found that in patients who developed cGvHD, there was a significant reduction in the abundance of the Eurobactereaceae family and Roseruria genus compared to the microbiota composition examined during the time of allo-HSCT. Evidence linking gut microbial disruption with the pathophysiology, progression, and prognosis of cGvHD, is scarce. Gut microbiota are closely associated with the pathophysiology of chronic autoimmune diseases [34], and as such, it is expected that changes in the gut microbial community are linked to the development of cGvHD. Shimizu et al. [35] studied the changes occurring in ocular microbial diversity in patients who underwent allo-HSCT and developed ocular cGvHD. Results showed that, in patients with ocular cGvHD, there was an increase in the abundance of both commensal and pathogenic bacteria compared to patients with no cGvHD and healthy controls. To our knowledge, our study is one of the few to assess specific alterations in gut microbial composition in patients with cGvHD. Hino et al. [36] prospectively assessed 59 patients who underwent allo-HSCT over a median follow-up time of 6.4 years. Of them, 23 developed cGvHD and alpha and beta diversity analyses did not differ compared with patients without cGvHD. Interestingly, an abundance of Faecalibacterium decreased in patients with extensive cGvHD compared with patients with limited or no cGvHD, suggesting that microbial perturbations may be associated with varying disease severity. Since cGvHD can affect up to 40% of patients undergoing allo-HSCT [37] and is also associated with inferior overall survival and higher treatment-related mortality [38], it is imperative to understand to what extent gut microbial composition can affect the incidence and clinical outcomes in this patient population.

Regarding the evolution of alpha diversity metrics over time, we observed that in patients with aGvHD, alpha diversity progressively increased over time. This observation was expected, since our patient population included by design patients who survived after the transplant, suggesting a rather positive outcome for the majority of patients. Also, although aGvHD did not resolve, it was controlled under treatment. In patients with cGvHD, on the other hand, alpha diversity eventually declined compared to the diversity observed at the time of allo-HSC, with the disease remaining stable after appropriate treatment. However, changes in alpha diversity were not statistically significant in both acute and chronic GvHD cases. Prospective studies that will conduct similar analyses in larger samples by taking into consideration the severity of symptoms and interim clinical events are needed to delineate the microbial community changes over time that might contribute to differential outcomes in patients with GvHD.

We acknowledge some limitations in our study. First, according to the study design, we included only patients that survived until day 180 following allo-HSCT and, as such, we could not comment on whether changes in the intestinal microbiome in patients that develop GvHD are linked to improved or worse survival outcomes. Next, this was a single-center study, and thus our results, may not be generalizable to other centers and institutions. This was a descriptive study, and our purpose was to document the changes in gut microbiome composition in patients who developed GvHD after allo-HSCT. We did not correlate our results with clinical outcomes of interest, such as mortality rate. Although there is clear evidence from previous studies that the choice of antimicrobial therapy affects the gut microbiome composition following allo-HSCT [39], we did not perform subgroup analyses based on antimicrobial therapy, due to the small sample size used in our analysis. Also, data regarding the microbiological composition in patients who underwent allo-HSCT and did not develop acute and/or chronic GvHD were not available in our study. As such, we emphasize the importance of conducting prospective studies that will delineate the composition of the intestinal microbial communities in patients who undergo allo-HSCT and develop GvHD, versus those who do not develop this complication. Also, although we acknowledge that intestinal GvHD could have impacted the results of our analysis, subgroup analysis based on the presence or absence of this disease entity was not possible due to the small sample size. Finally, due to the small sample size, our patient population did not allow for multivariate analysis of potentially confounding variables, including prior radiotherapy, antifungal/antibacterial prophylaxis, therapeutic interventions for GvHD, bacterial infection, and episodes of colitis and/or mucositis. Nevertheless, since antibacterial/antifungal prophylaxis was similar to all patients, we do not believe that they could play an important role. Similarly, we did not observe severe mucositis in these patients.

## 4. Materials and Methods

### 4.1. Study Design

We conducted a single-center, prospective study including patients ≥ 18 years of age who underwent allo-HSCT from matched related or unrelated donors at our JACIE (Joint Accreditation Committee—International Society for Cellular Therapy [ISCT] and European Society for Blood and Marrow Transplantation [EBMT]) accredited Unit of Papanikolaou Hospital, from January to June 2020. This study was approved by our Institutional Review Board at Papanikolaou Hospital (protocol number 126/2019, date of approval 2 May 2022). All patients provided written informed consent in accordance with the Helsinki Declaration and were subsequently enrolled in a fecal collection protocol. Fecal biospecimens were collected at days −2 to +2 (timepoint 1), +11 to +17 (timepoint 2), +25 to +30 (timepoint 3), +90 (timepoint 4), and +180 (timepoint 5), with day 0 being the day of allo-HSCT (analysis of fecal specimens is provided in Supplementary Methods). Patients were followed up for a minimum of 6 months after the last sample was collected.

### 4.2. Study Endpoint

As an endpoint, we sought to investigate the microbial abundance of taxa at the phylum, family, genus, and species level between the various timepoints of fecal specimen collection in patients who developed aGvHD and cGvHD after allo-HSCT.

### 4.3. Study Population

We included patients who were at least 18 years old who underwent allo-HSCT during the study period (January–June 2020). We included patients who survived until Day 180 (timepoint 5) following allo-HSCT. Enrolled patients had a diagnosis of hematologic malignancy and received myeloablative or nonmyeloablative conditioning regimens according to the standard practice of the JACIE-accredited center. Specifically, conditioning regimens were administered according to the Department’s protocol [40]. Myeloablative conditioning for fit patients includes busulfan and cyclophosphamide (BuCy), whereas reduced toxicity conditioning for unfit patients includes treosulfan plus fludarabine (FT14) [41]. Nonmyeloablative conditioning included fludarabine-melphalan (FluMel) for lymphoid malignancies. Patients had an available sibling or matched or mismatched unrelated donor (based on molecular typing of HLA-A, -B, -C, -DR, and -DQ).

Only patients with grade II or higher aGvHD, according to the Glucksberg criteria [42] and patients with moderate or severe cGvHD based on the NIH consensus criteria [43], were included in the study. Patients who developed both acute and chronic GvHD were considered to have cGvHD. 

Exclusion criteria included age of participants < 18 years, refusal to provide written informed consent, insufficient number of fecal specimens for analysis, grade I aGvHD and mild cGvHD.

Patients were given antimicrobial prophylaxis when the absolute neutrophil count was <500 cells/mm^3^, or by day −1, the day before stem cell infusion, and continued until neutrophil engraftment (defined as the first day of achieving a neutrophil count > 500 cells/mm^3^ for 3 consecutive days after allo-HSCT). When patients met the criteria for neutropenic fever, a fever workup was performed, including 2 sets of blood cultures, urinalysis, urine culture, and CT scan; antibiotics were escalated (i.e., antipseudomonal beta-lactam and vancomycin), and the prophylactic agent was discontinued. Standard antibiotic prophylaxis at our institution includes fluoroquinolone (500 mg of oral ciprofloxacin twice a day) or rifaximin (200 mg twice a day), according to the treating physician’s judgment, for anticipated profound and prolonged neutropenia.

Baseline demographic data, underlying hematologic disease characteristics, laboratory tests, treatment about GvHD, including the number of patients who developed aGvHD vs. cGvHD, grade, and therapeutic regimen for GvHD, were retrieved from medical records.

### 4.4. Statistical Analysis

We assessed differences in microbiota composition and diversity between timepoint 4 and timepoint 1 as the primary endpoints of our study since scarce data exist beyond timepoint 4 in allo-HSCT. Patient recruitment was limited due to COVID-19 restraints and increased requirements of the study protocol. Therefore, we performed a post hoc power analysis for differences between timepoint 4 and timepoint 1 confirming that the size of 20 patients is adequate for a power of 80% with the level of significance at 0.05.

Data analysis was performed using the program R Statistical Computing (R statistics software version 4.1.2, R Foundation for Statistical Computing, Vienna, Austria). Descriptive statistics were performed using median and range for continuous variables and frequency for categorical variables. Continuous variables were assessed for normality and compared using a *t*-test or Mann–Whitney test. Mean values (with standard deviation) were used in the case of normal distribution and the median values (with the interquartile range) were used in the case of non-normal distribution. Categorical variables were compared using a chi-square test. All tests were two-sided. The level of statistical significance was set at 0.05. We used 95% confidence intervals where appropriate. 

To conduct statistical analysis of the microbiome data, open source R programming language 4.1.2.v (R Core Team, New Zealand, Australia) was used [44]. Vegan 2.5.3v [45] and phyloseq 1.24.2v [46] R packages (R Core Team, New Zealand, Australia) were imported for implementation of the data analysis and ggplot2 3.1.0v [47] package (R Core Team, New Zealand, Australia) for data visualization. The relative abundance of taxa at phylum, family, genus, and species level, was explored with the Kruskal–Wallis non-parametric test, bar plots, heatmap plots and Venn diagrams. 

Richness was evaluated and Shannon and Inverse Simpson alpha diversity indexes were calculated for each timepoint per group (aGVHD and cGVHD), in order to explore the microbial diversity within each sample. Non-parametric Kruskal–Wallis test was also implemented to detect differences in alpha diversity indices among timepoints per group and among the groups in specific timepoints. Additionally, to further explore our data, linear discriminant analysis Effect Size (LEfSe) method [48] was applied to detect differentially abundant taxa, with threshold LDA value 2.0, between controls and patients. 

To consider beta diversity between samples, the Bray–Curtis dissimilarity matrix was calculated, and Principal Coordinate Analysis (PCoA) was applied to find out taxa that were counted more in the total variance and for pattern visualization. To confirm significant differences between patients and controls and between subgroups of patients, Permutational Multivariate Analysis of Variance (PERMANOVA) [49] with 1000 permutations was implemented together with PERMutational analysis of multivariate DISPersion (PERMDISP) [49] to further strengthen the results of PERMANOVA test (Supplementary Figures S9 and S10).

## 5. Conclusions

In this study, we provide additional insights regarding changes in gut microbiota composition occurring in patients undergoing allo-HSCT who developed GvHD. Our study reinforces the results of previous analyses, but at the same time provides novel clues regarding the specific gut microbial perturbations in patients who developed cGvHD, a disease entity not thoroughly elucidated with regard to the role of the microbiome in its pathogenesis. Future studies will need to clinically correlate these specific changes in gut microbiota and incorporate additional variables in their analyses, including choice of myeloablative regimen and/or antimicrobial prophylaxis, in order to delineate their effect in the gut microbial community. Since aGvHD and cGvHD are considered disease entities within a spectrum of disease (graft-versus-host disease), it is speculated that the intestinal microbiome is involved in different ways in its pathogenesis. In order to provide a more comprehensive explanation regarding the differences in intestinal microbiota between acute and chronic GvHD, future studies are required that will consider larger sample size and more sophisticated methods for analyzing the microbiota composition. The aim of our study was to provide a descriptive analysis of the microbiota in patients with aGvHD and cGvHD, and, as such, we do not possess sufficient data to propose plausible explanations regarding the differences observed between the groups. Future research, especially in the field of chronic GvHD, including new advances in the therapeutic landscape, is urgently needed to tackle this challenge in transplantation.

## Figures and Tables

**Table 1:** Baseline characteristics of patients included in the study.

Characteristics	aGVHD	cGVHD	GVHD Total
Total patients	8 (40)	12 (60)	20 (100)
Sex (male)	5 (25)	9 (45)	14 (70)
Median patient age	50	52.6	51.3
Donor			
Related	3 (15)	7 (35)	10 (50)
Unrelated (matched)	4 (20)	3 (15)	7 (35)
Unrelated (mismatched)	1 (5)	2 (10)	3 (15)
Graft source			
Peripheral blood stem cell	7 (35)	11 (55)	18 (90)
Bone marrow	1 (5)	1 (5)	2 (10)
aGVHD Glucksberg scale			
Grade II	3 (37.5)	N/A	N/A
Grade III	5 (62.5)	N/A	N/A
cGvHD severity			
Moderate	N/A	8 (66.7)	N/A
Severe	N/A	4 (33.4)	N/A
Underlying disease			
Acute myeloid leukemia	3 (15)	5 (25)	8 (40)
Acute lymphoblastic leukemia	3 (15)	3 (15)	6 (30)
Chronic myelogenous leukemia	2 (10)	2 (10)	4 (20)
Myelodysplastic syndrome	0 (0)	2 (10)	2 (10)
HCT—CI *	2.3	2.6	2.5
EBMT score *	3.5	3.6	3.6
DRI			
Intermediate	3 (15)	5 (25)	8 (40)
High	5 (25)	7 (35)	12 (60)
Conditioning regimen			
Myeloablative	2 (10)	4 (20)	6 (30)
Non-myeloablative	6 (30)	8 (40)	14 (70)

Results are number (percentage, %), unless specified otherwise. * Mean values are reported. Abbreviations list: aGVHD: acute graft-versus-host disease; cGVHD: chronic graft-versus-host disease; GVHD: graft-versus-host disease; DRI: disease risk index; EBMT: European Group for Blood and Marrow Transplantation; HCT-CI: hematopoietic cell transplantation—comorbidity index; N/A: not available.

**Table 2:** Abundance analysis at the phylum, family, genus, and species levels of the 10 most abundant taxa in patients with aGVHD at timepoints 1 and 4.

Level of Analysis	Timepoint 1 *	Level of Analysis	Timepoint 4 *
Phylum	Total Abundance (%)	Phylum	Total Abundance (%)
*Bacteroidetes*	62.43	*Firmicutes*	75.73
*Firmicutes*	22.42	*Bacteroidetes*	18.41
*Proteobacteria*	14.22	*Proteobacteria*	4.07
*Actinobacteria*	0.92	*Actinobacteria*	1.75
*Verrucomicrobia*	0	*Verrucomicrobia*	0.05
**Family**		**Family**	
*Bacteroidaceae*	41.69	*Erysipelotrichaceae*	18.65
*Porphyromonadaceae*	19.94	*Bacteroidaceae*	17.91
*Enterobacteriaceae*	13.3	*Lachnospiraceae*	16.38
*Clostridiaceae*	4.79	*Enterococcaceae*	11.4
*Lachnospiraceae*	4.57	*Streptococcaceae*	8.92
*Acidaminococcaceae*	3.38	*Ruminococcaceae*	5.77
*Erysipelotrichaceae*	3.15	*Clostridiaceae*	5.22
*Ruminococcaceae*	2.94	*Lactobacillaceae*	2.86
*unclassified Clostridiales*	1.76	*Hyphomicrobiaceae*	2.73
*Veillonellaceae*	1.21	*Aerococcaceae*	1.95
**Genus** (**N**) **		**Genus** (**N**) **	
*Bacteroides* (6)	50.75	*Clostridium* (7)	24.62
*Parabacteroides* (5)	23.66	*Bacteroides* (5)	19.45
*Clostridium* (8)	6.21	*Enterococcus* (8)	12.79
*Phascolarctobacterium* (1)	2.22	*Streptococcus* (5)	9.91
*Flavonifractor* (5)	2.06	*Ruminococcus* (4)	4.66
*Acidaminococcus* (1)	2.03	*Ruminococcus* (3)	4.19
*Faecalibacterium* (5)	1.38	*Faecalibacterium* (2)	3.6
*Eubacterium* (4)	1.25	*Lactobacillus* (4)	3.31
*Ruminococcus* (5)	1.1	*Lachnoclostridium* (3)	3.25
*Alistipes* (4)	1.01	*Gemmiger* (1)	3.17
**Species** (**N**) **		**Species** (**N**) **	
*Bacteroides vulgatus* (4)	33.86	*Clostridium ramosum* (4)	11.56
*Parabacteroides merdae* (4)	23.66	*Clostridium spiroforme* (4)	9.2
*Bacteroides dorei* (1)	8.13	*Streptococcus thermophilus* (3)	8.24
*Bacteroides thetaiotaomicron* (4)	3.38	*Bacteroides vulgatus* (5)	5.79
*Bacteroides uniformis* (4)	3.05	*Ruminococcus gnavus* (3)	5.47
*Bacteroides coprocola* (1)	2.8	*Bacteroides intestinalis* (3)	5.44
*Phascolarctobacterium faecium* (1)	2.53	*Faecalibacterium prausnitzii* (1)	4.59
*Acidaminococcus intestine* (1)	2.34	*Gemmiger formicilis* (1)	4.19
*Flavonifractor plautii* (5)	2.1	*Lactobacillus fermentum* (2)	4.09
*Eubacterium cylindroides* (1)	1.36	*Ruminococcus gnavus* (4)	4.08

* Timepoint 1 occurred between days −2 and +2, and timepoint 4 occurred at day +90, with day 0 being the day of stem cell infusion. ** N denotes the number of patients, in whom specific genus-level bacteria or bacterial species were isolated (i.e., prevalence). Abbreviations list: aGVHD: acute graft-versus-host disease; N: number.

**Table 3:** Abundance analysis at the phylum, family, genus, and species levels of the 10 most abundant taxa in patients with cGVHD at timepoints 1 and 4.

Level of Analysis	Timepoint 1 *	Level of Analysis	Timepoint 4 *
Phylum	Total Abundance (%)	Phylum	Total Abundance (%)
*Bacteroidetes*	50.31	*Firmicutes*	65.85
*Firmicutes*	35.06	*Bacteroidetes*	26.37
*Proteobacteria*	13.54	*Proteobacteria*	7.55
*Actinobacteria*	1.07	*Actinobacteria*	0.14
*Verrucomicrobia*	0.01	*Deferribacteres*	0.05
		*Verrucomicrobia*	0.03
**Family**		**Family**	
*Bacteroidaceae*	34.96	*Bacteroidaceae*	25.67
*Porphyromonadaceae*	14.76	*Erysipelotrichaceae*	17.77
*Lachnospiraceae*	13.82	*Clostridiaceae*	12.69
*Enterobacteriaceae*	11.84	*Lachnospiraceae*	11.66
*Clostridiaceae*	7.37	*Ruminococcaceae*	8.97
*Erysipelotrichaceae*	3.29	unclassified *Clostridiales*	7.21
*Acidaminococcaceae*	2.64	*Enterobacteriaceae*	5.06
unclassified *Clostridiales*	1.9	*Enterococcaceae*	2.4
*Ruminococcaceae*	1.79	*Oscillospiraceae*	1.51
*Eubacteriaceae*	1.32	*Hyphomicrobiaceae*	1.38
**Genus** (**N**) **		**Genus** (**N**) **	
*Bacteroides* (9)	43.24	*Bacteroides* (9)	32.87
*Parabacteroides* (6)	17.59	*Clostridium* (10)	31.84
*Clostridium* (12)	9.61	*Flavonifractor* (7)	9.36
*Roseburia* (6)	3.97	*Ruminococcus* (6)	3.37
*Eubacterium* (6)	2.53	*Ruminococcus* (6)	3.07
*Lachnoclostridium* (8)	2.47	*Enterococcus* (8)	2.92
*Ruminococcus* (9)	2.35	*Faecalibacterium* (4)	2.52
*Flavonifractor* (8)	2.21	*Lachnoclostridium* (4)	2.24
*Ruminococcus* (8)	2.07	*Subdoligranulum* (6)	2.05
*Phascolarctobacterium* (2)	1.71	*Gemmiger* (1)	1.82
**Species** (**N**) **		**Species** (**N**) **	
*Bacteroides vulgatus* (9)	35.35	*Bacteroides vulgatus* (8)	22.95
*Parabacteroides merdae* (5)	18.36	*Clostridium ramosum* (3)	14.39
*Roseburia intestinalis* (4)	3.48	*Flavonifractor plautii* (7)	11.08
*Bacteroides coprocola* (2)	2.71	*Clostridium spiroforme* (5)	7.82
*Bacteroides thetaiotaomicron* (5)	2.52	*Clostridium aldenense* (5)	5.45
*Bacteroides uniformis* (5)	2.5	*Ruminococcus gnavus* (5)	3.73
*Flavonifractor plautii* (8)	2.4	*Bacteroides intestinalis* (4)	2.96
*Lachnoclostridium clostridioforme* (4)	2.37	*Ruminococcus gnavus* (6)	2.79
*Bacteroides fragilis* (3)	2.18	*Faecalibacterium prausnitzii* (2)	2.51
*Clostridium spiroforme* (8)	2.04	*Subdoligranulum* spp. (5)	2.29

* Timepoint 1 occurred between days −2 and +2, and timepoint 4 occurred at day +90, with day 0 being the day of stem cell infusion. ** N denotes the number of patients, in whom specific genus-level bacteria or bacterial species were isolated (i.e., prevalence). Abbreviations list: aGVHD: acute graft-versus-host disease; N: number.

## Data Availability

Data for this study is available and can be provided upon reasonable request.

## Supplementary Materials

The following supporting information can be downloaded at: https://www.mdpi.com/article/10.3390/ijms25115789/s1. Reference [50] is cited in the supplementary materials.
