# Supplementary material for: Alteration of Gut Microbiota Composition and Diversity in Acute and/or Chronic Graft-versus-Host Disease Following Hematopoietic Stem Cell Transplantation: A Prospective Cohort Study
Source: Int J Mol Sci. 2024 May 26;25(11):5789. doi: 10.3390/ijms25115789 (PMC11171546; doi:10.3390/ijms25115789)
Supplement: Supplementary file 1 [file ijms-25-05789-s001.zip › ijms-3001577-supplementary.pdf]

Supplementary Material

Supplement to: Alteration of Gut Microbiota Composition and Diversity in Acute and/or Chronic Graft-versus-Host-Disease Following Hematopoietic Stem Cell Transplantation: A Prospective Cohort Study

1 Supplementary Data

<u>**Supplementary Methods**</u>

Analysis of specimens

Primary stool samples of the patients were pretreated according to the procedure protocol of the Project of the Human Microbiome of the National Health Organization and in the final product, genetic isolation was performed with the DNeasy PowerSoil Kit (QIAGEN). Isolated DNA was used for PCR procedure using the Ion 16S ™ Metagenomics Kit (Life Technologies) to amplify the entire 16S portion of the microbiome and the PCR product was purified with AMPure XP reagent (Agencourt). Ion Torrent-compatible bar-coded adapters were ligated to the sheared DNA using the Ion Xpress Plus fragment library kit (Life Technologies). The bar-coded libraries were pooled and purified with AMPure XP reagent (Agencourt). Quantitative PCR was performed on the pooled bar-coded libraries to assess the quality and to determine the template dilution factor for emulsion PCR. The pool was diluted appropriately and amplified on Ion Sphere particles (ISPs) using the Ion OneTouch instrument (Life Technologies). The pool of viral libraries was enriched for template-positive ISPs on the Ion OneTouch ES instrument (Life Technologies). Sequencing was performed on the Ion Torrent PGM using Ion 316 chips (1).

2 Supplementary Tables and Figures

Table S1: Summary of sequencing and microbiome profiling in the study.

Table S2: Ranges of median values of alpha-diversity indices measured with the Shannon and InvSimpson indices in patients with aGvHD and cGvHD.

Figure S1: Abundance analysis in the form of bar charts for the phyla included in the analysis of patients with acute GvHD between timepoint 1 (-2 to +2 days after transplantation) and timepoint 4 (90 days following transplantation) in patients with acute GvHD. Abbreviation: GvHD: graft-versus-host disease.

Figure S2: Abundance analysis in the form of bar charts for the totality of families (A) and the ten most prevalent families (B) included in the analysis of patients with acute GvHD between timepoint 1 (-2 to +2 days after transplantation) and timepoint 4 (90 days following transplantation) in patients with acute GvHD. Abbreviation: GvHD: graft-versus-host disease.

Figure S3: Abundance analysis in the form of bar charts for the totality of genera (A) and the ten most prevalent genera (B) included in the analysis of patients with acute GvHD between timepoint 1 (-2 to +2 days after transplantation) and timepoint 4 (90 days following transplantation) in patients with acute GvHD. Abbreviation: GvHD: graft-versus-host disease.

Figure S4: Abundance analysis in the form of bar charts for the totality of species (A) and the ten most prevalent species (B) included in the analysis of patients with acute GvHD between timepoint 1 (-2 to +2 days after transplantation) and timepoint 4 (90 days following transplantation) in patients with acute GvHD. Abbreviation: GvHD: graft-versus-host disease.

Figure S5: Abundance analysis in the form of bar charts for phyla included in the analysis of patients with acute GvHD between timepoint 1 (-2 to +2 days after transplantation) and timepoint 4 (90 days following transplantation) in patients with chronic GvHD. Abbreviation: GvHD: graft-versus-host disease.

Figure S6: Abundance analysis in the form of bar charts for the totality of families (A) and the ten most prevalent families (B) included in the analysis of patients with chronic GvHD between timepoint 1 (-2 to +2 days after transplantation) and timepoint 4 (90 days following transplantation) in patients with acute GvHD. Abbreviation: GvHD: graft-versus-host disease.

Figure S7: Abundance analysis in the form of bar charts for the totality of genera (A) and the ten most prevalent genera (B) included in the analysis of patients with chronic GvHD between timepoint 1 (-2 to +2 days after transplantation) and timepoint 4 (90 days following transplantation) in patients with acute GvHD. Abbreviation: GvHD: graft-versus-host disease.

Figure S8: Abundance analysis in the form of bar charts for the totality of species (A) and the ten most prevalent species (B) included in the analysis of patients with chronic GvHD between timepoint 1 (-2 to +2 days after transplantation) and timepoint 4 (90 days following transplantation) in patients with acute GvHD. Abbreviation: GvHD: graft-versus-host disease.

Figure S9: PCoA based on the overall structure of the gut microbial community at the genus level in patients with aGVHD between timepoint 1 and timepoint 4. PC1 (x-axis) could explain 21.1% and PC2 (y-axis) could explain 20% of the difference in the abundance of bacteria at the genus level between timepoint 1 and timepoint 4, with differences in the microbial communities between timepoint 1 and timepoint 4 not being statistically significant (P = 0.09). Abbreviations list: aGVHD: acute graft-versus-host-disease; PCoA: principal coordinate analysis.

Figure S10: PCoA based on the overall structure of the gut microbial community at the genus level in patients with aGVHD between timepoint 1 and timepoint 4. PC1 (x-axis) could explain 20.8% and PC2 (y-axis) could explain 18% of the difference in the abundance of bacteria at the genus level between timepoint 1 and timepoint 4, with results, though, not being statistically significant (P =0.60). Abbreviations list: cGVHD: chronic graft-versus-host-disease; PCoA: principal coordinate analysis.

Table S1: Summary of sequencing and microbiome profiling in the study.

Taxonomic Level	OTUs detected	Total Reads	Mean Reads
Phylum Level	11 phyla	3.099.730	281.794
Family Level	99 families	3.099.730	31.310
Genus Level	148 genera	2.456.647	24.814
Species Level	285 species	1.786.725	18.047

Abbreviations: OTUs: Operational taxonomic units.

Table S2: Ranges of median values of alpha-diversity indices measured with the Shannon and InvSimpson indices in patients with aGvHD and cGvHD.

aGVHD: Ranges of a-diversity indices						
	Timepoint1	Timepoint2	Timepoint3	Timepoint4	Timepoint5	Timepoint6
Shannon	1.41	2.46	1.72	1.61	1.79	0.09
IvnSimpson	1.52	5.21	4.90	4.42	5.24	0.01

cGVHD: Ranges of a-diversity indices						
	Timepoint1	Timepoint2	Timepoint3	Timepoint4	Timepoint5	Timepoint6
Shannon	1.65	2.30	1.74	1.76	1.61	0.89
IvnSimpson	5.08	7.06	3.91	4.48	5.05	2.87

Abbreviations: aGvHD: acute graft-versus-host disease; cGvHD: chronic graft-versus-host disease.

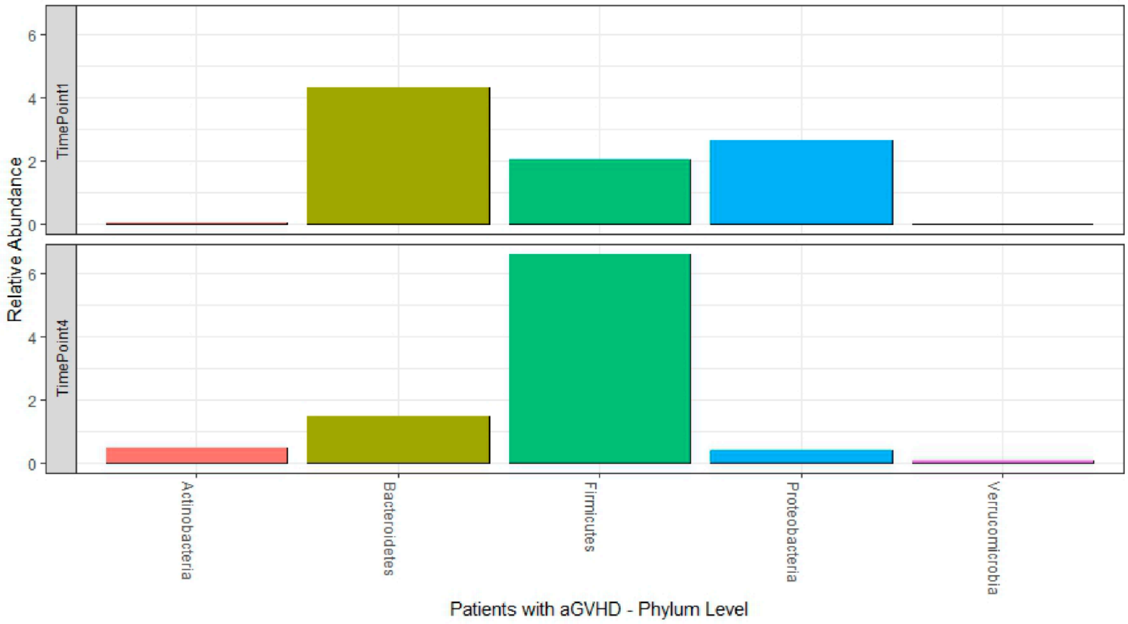

Figure S1: Abundance analysis in the form of bar charts for the phyla included in the analysis of patients with acute GvHD between timepoint 1 (-2 to +2 days after transplantation) and timepoint 4 (90 days following transplantation) in patients with acute GvHD. Abbreviation: GvHD: graft-versus-host disease.

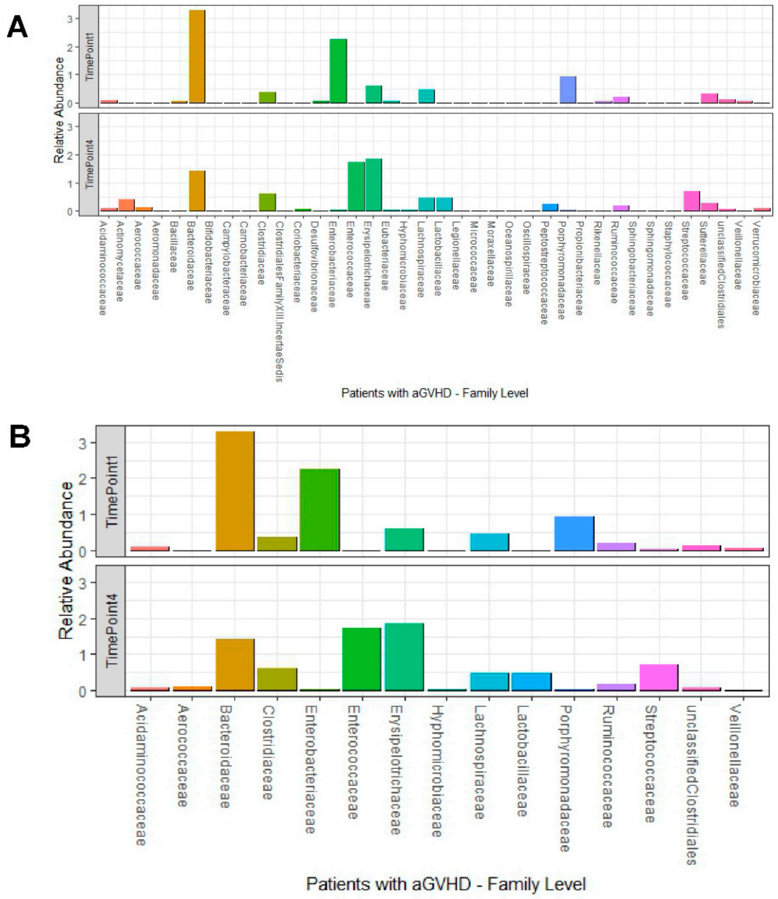

Figure S2: Abundance analysis in the form of bar charts for the totality of families (A) and the ten most prevalent families (B) included in the analysis of patients with acute GvHD between timepoint 1 (-2 to +2 days after transplantation) and timepoint 4 (90 days following transplantation) in patients with acute GvHD. Abbreviation: GvHD: graft-versus-host disease.

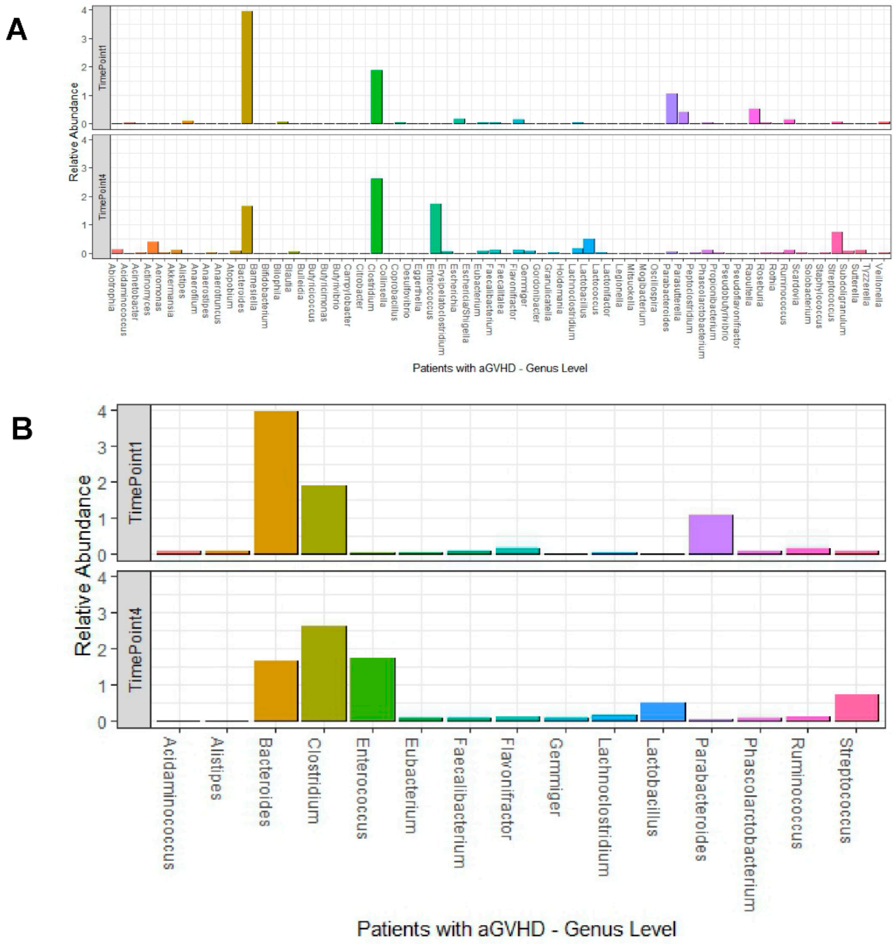

Figure S3: Abundance analysis in the form of bar charts for the totality of genera (A) and the ten most prevalent genera (B) included in the analysis of patients with acute GvHD between timepoint 1 (-2 to +2 days after transplantation) and timepoint 4 (90 days following transplantation) in patients with acute GvHD. Abbreviation: GvHD: graft-versus-host disease.

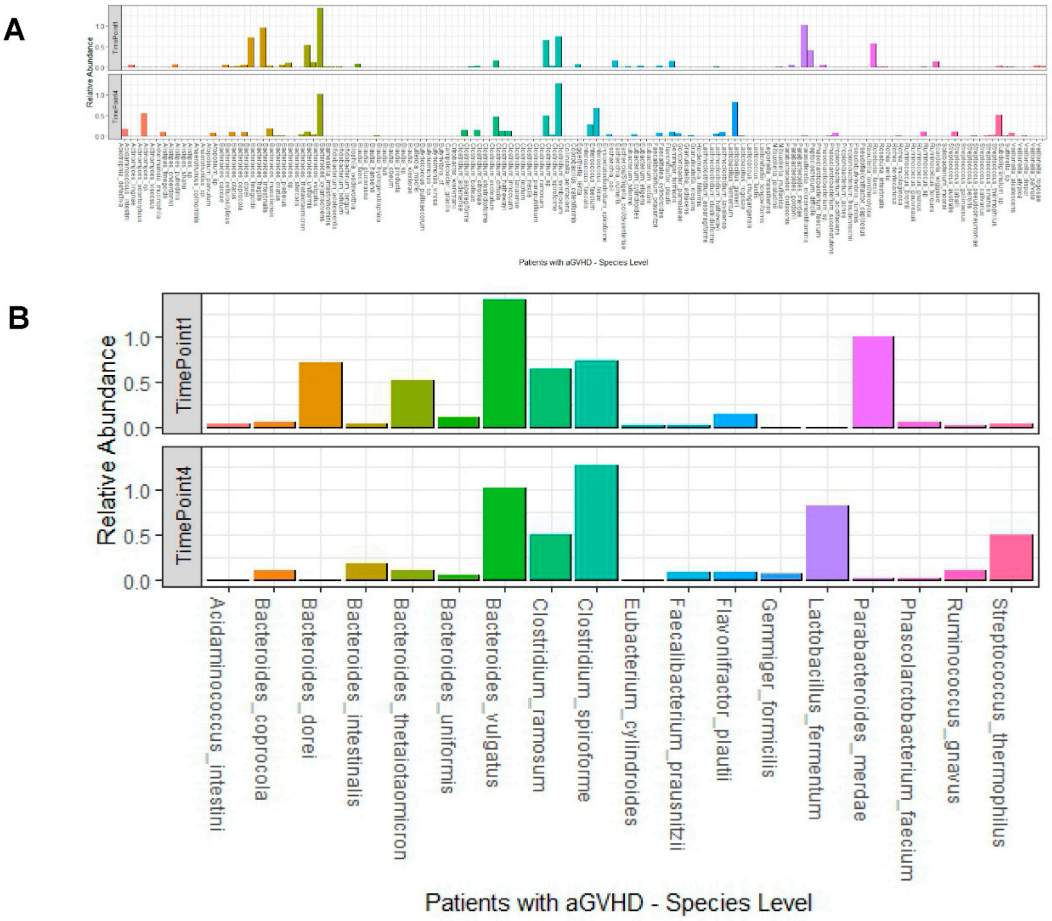

Figure S4: Abundance analysis in the form of bar charts for the totality of species (A) and the ten most prevalent species (B) included in the analysis of patients with acute GvHD between timepoint 1 (-2 to +2 days after transplantation) and timepoint 4 (90 days following transplantation) in patients with acute GvHD. Abbreviation: GvHD: graft-versus-host disease.

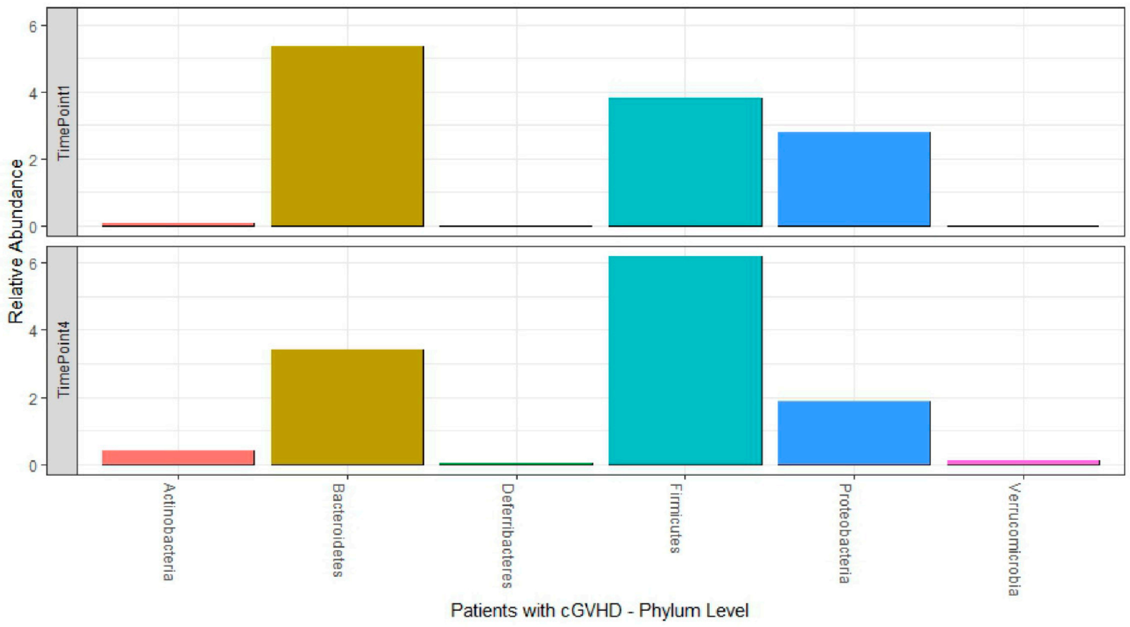

Figure S5: Abundance analysis in the form of bar charts for phyla included in the analysis of patients with acute GvHD between timepoint 1 (-2 to +2 days after transplantation) and timepoint 4 (90 days following transplantation) in patients with chronic GvHD. Abbreviation: GvHD: graft-versus-host disease.

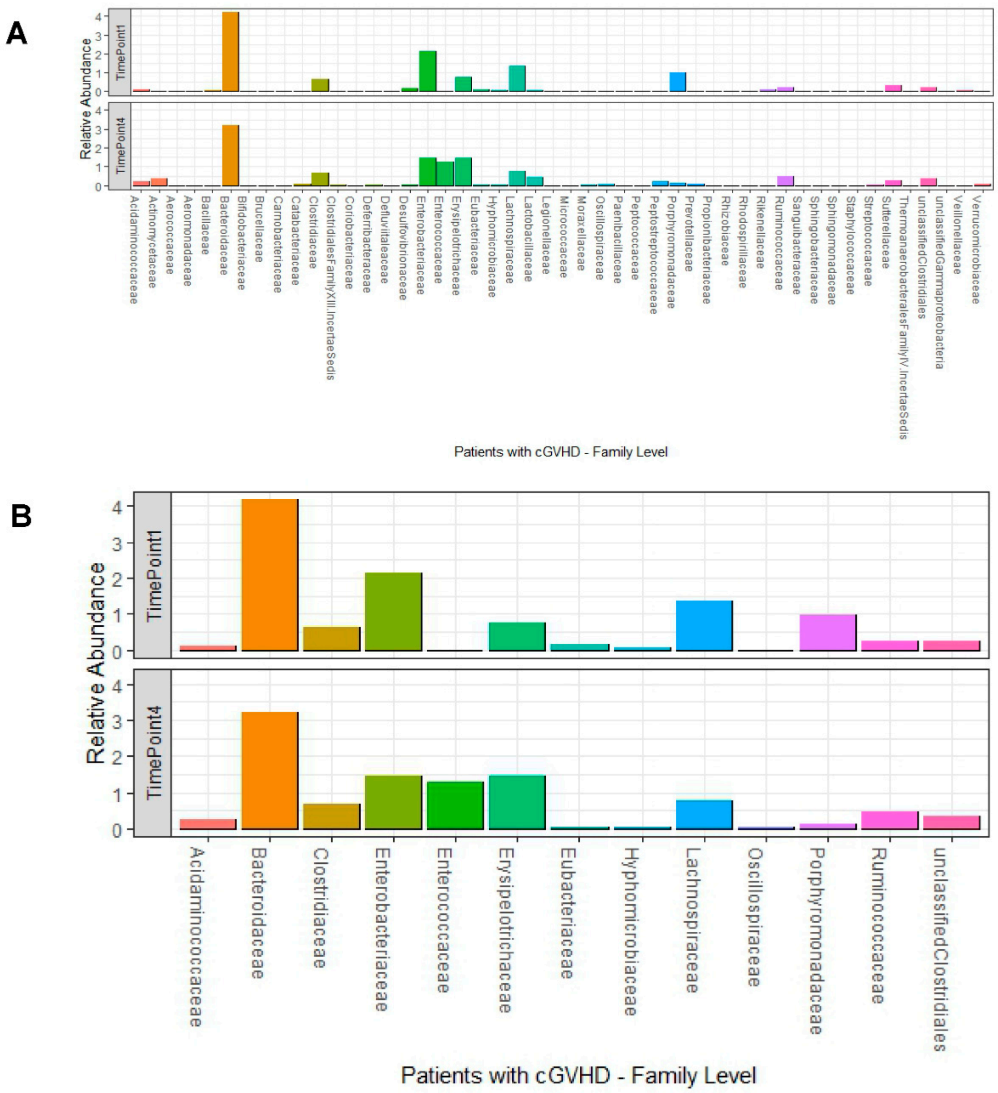

Figure S6: Abundance analysis in the form of bar charts for the totality of families (A) and the ten most prevalent families (B) included in the analysis of patients with chronic GvHD between timepoint 1 (-2 to +2 days after transplantation) and timepoint 4 (90 days following transplantation) in patients with acute GvHD. Abbreviation: GvHD: graft-versus-host disease.

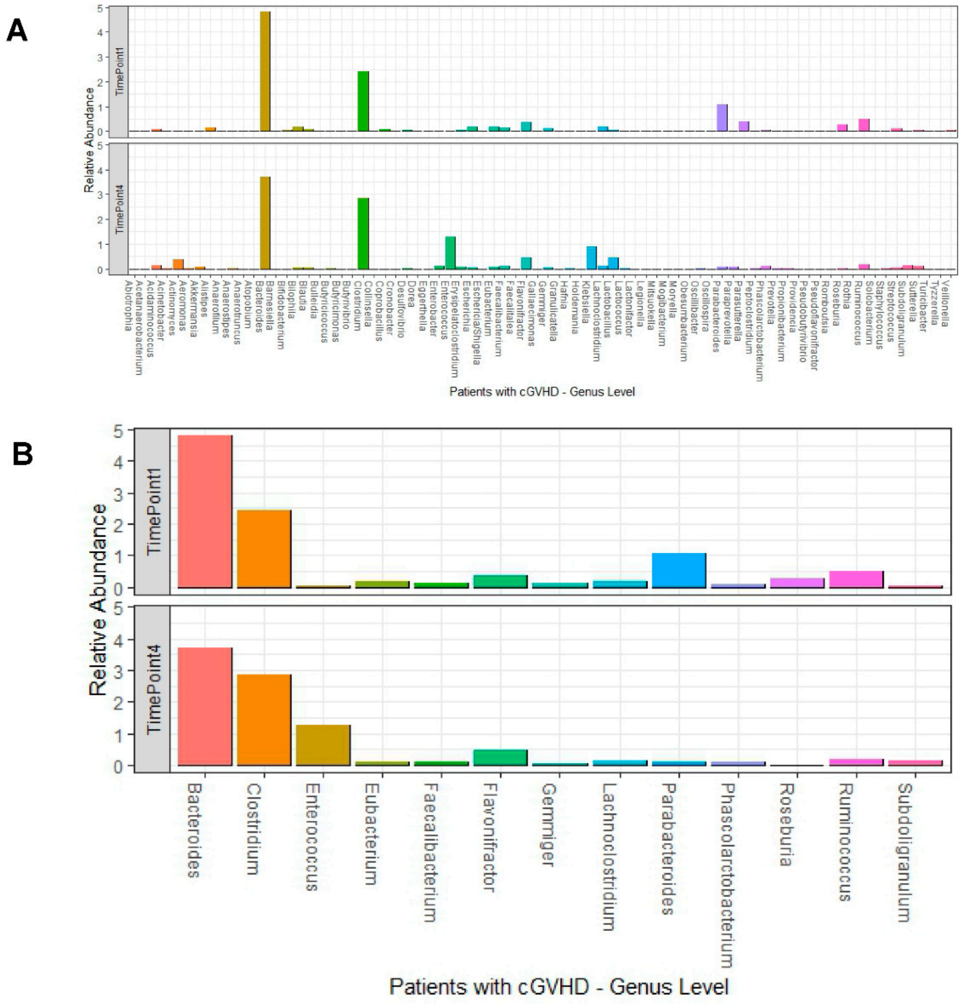

Figure S7: Abundance analysis in the form of bar charts for the totality of genera (A) and the ten most prevalent genera (B) included in the analysis of patients with chronic GvHD between timepoint 1 (-2 to +2 days after transplantation) and timepoint 4 (90 days following transplantation) in patients with acute GvHD. Abbreviation: GvHD: graft-versus-host disease.

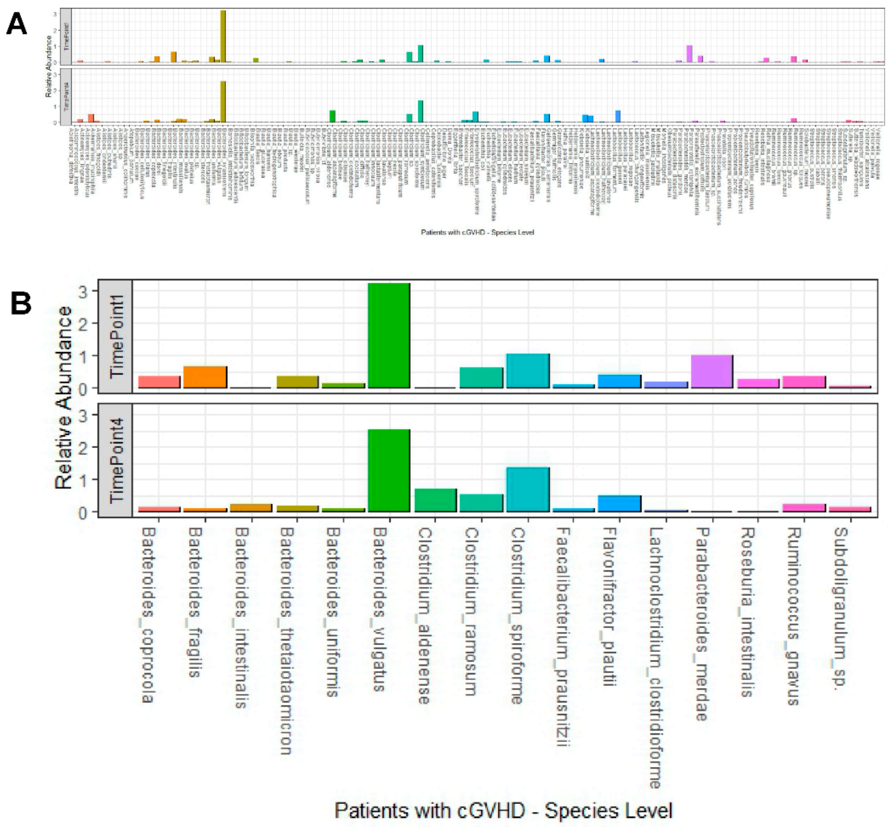

Figure S8: Abundance analysis in the form of bar charts for the totality of species (A) and the ten most prevalent species (B) included in the analysis of patients with chronic GvHD between timepoint 1 (-2 to +2 days after transplantation) and timepoint 4 (90 days following transplantation) in patients with acute GvHD. Abbreviation: GvHD: graft-versus-host disease.

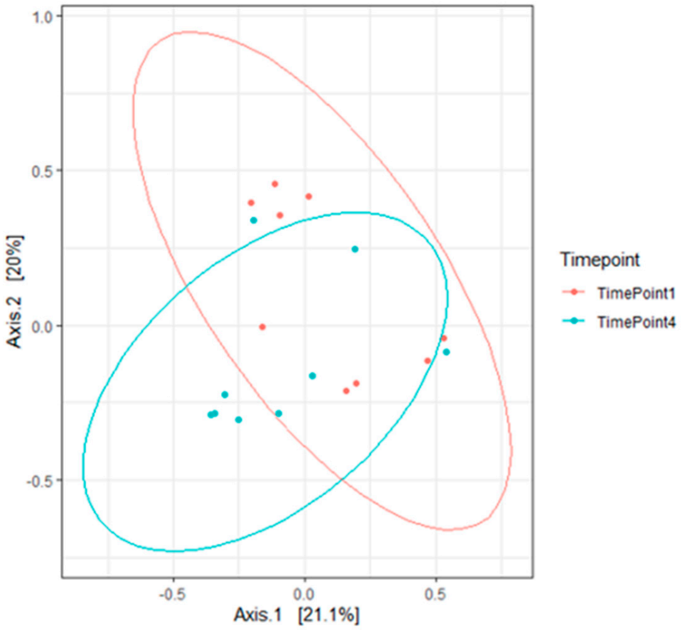

Figure S9: PCoA based on the overall structure of the gut microbial community at the genus level in patients with aGVHD between timepoint 1 and timepoint 4. PC1 (x-axis) could explain 21.1% and PC2 (y-axis) could explain 20% of the difference in the abundance of bacteria at the genus level between timepoint 1 and timepoint 4, with differences in the microbial communities between timepoint 1 and timepoint 4 not being statistically significant (*P* = 0.09). Abbreviations list: aGVHD: acute graft-versus-host-disease; PCoA: principal coordinate analysis.

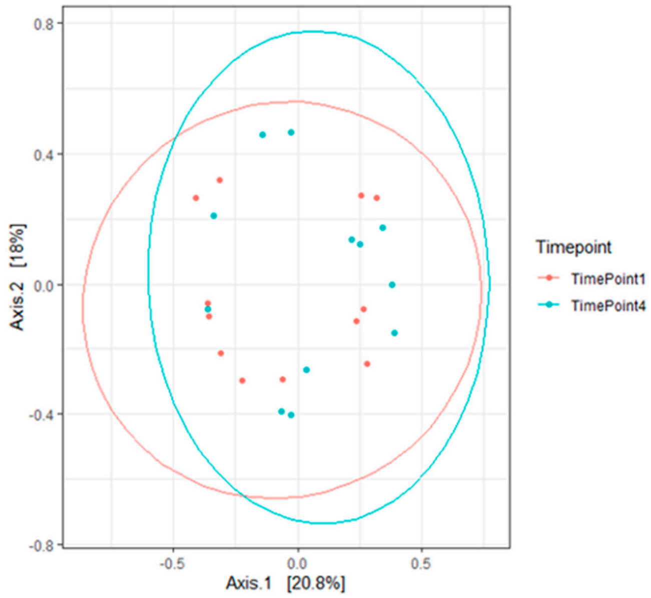

Figure S10: PCoA based on the overall structure of the gut microbial community at the genus level in patients with aGVHD between timepoint 1 and timepoint 4. PC1 (x-axis) could explain 20.8% and PC2 (y-axis) could explain 18% of the difference in the abundance of bacteria at the genus level between timepoint 1 and timepoint 4, with results, though, not being statistically significant (P =0.60). Abbreviations list: cGVHD: chronic graft-versus-host-disease; PCoA: principal coordinate analysis.

References

1. McInnes PaC, M., 2010. Manual of Procedures for Human Microbiome Project. [online] Hmpdacc.org. [Available from: https://hmpdacc.org/hmp/doc/HMP_MOP_Version12_0_072910.pdf. Accessed on October 10, 2023.
